# Advances for the Topographic Characterisation of SMC Materials

**DOI:** 10.3390/ma2031084

**Published:** 2009-08-27

**Authors:** Alfredo Calvimontes, Karina Grundke, Anett Müller, Manfred Stamm

**Affiliations:** Leibniz-Institute of Polymer Research Dresden, Hohe Strasse 6, 01069 Dresden, Germany; E-Mails: grundke@ipfdd.de (K.G.); mueller-anett@ipfdd.de (A.M.); stamm@ipfdd.de (M.S.)

**Keywords:** moulding compounds, surface properties, surface morphology, topographic characterisation

## Abstract

For a comprehensive study of Sheet Moulding Compound (SMC) surfaces, topographical data obtained by a contact-free optical method (chromatic aberration confocal imaging) were systematically acquired to characterise these surfaces with regard to their statistical, functional and volumetrical properties. Optimal sampling conditions (cut-off length and resolution) were obtained by a topographical-statistical procedure proposed in the present work. By using different length scales specific morphologies due to the influence of moulding conditions, metallic mould topography, glass fibre content and glass fibre orientation can be characterized. The aim of this study is to suggest a systematic topographical characterization procedure for composite materials in order to study and recognize the influence of production conditions on their surface quality.

## 1. Introduction 

Due to their light weight, good mechanical properties, corrosion resistance and design flexibility, fibre-reinforced composite materials are being increasingly used in the automotive industry. Materials commonly used for these applications are Sheet Moulding Compounds (SMC), which consist of glass fibres, unsaturated polyesters with a styrene matrix as co-monomer for crosslinking, inorganic fillers (mainly calcium carbonate) and other chemicals for curing, aspect control, etc.

Due to the high heterogeneity of SMC materials it is difficult to meet the optical requirements of the automotive industry. By controlling the processing conditions, surface properties of SMC can be optimized for subsequent coating. It was the aim of this study to perform a systematic topographical functional characterisation of SMC materials in order to qualify and quantify the coatability and the optical parameters of these surfaces. Only a small number of studies using SMC materials for compression moulding have shown the effects of formulation and moulding conditions on the resulting surface topography [1,2]. 

The aim of this investigation was not to suggest some optimal formulation or production conditions to obtain smoother SMC surfaces, but rather to present advances regarding their topographic characterisation and the better understanding of the relationships between production conditions and resulting surface morphologies. Heat interchange (cooling) and conditions of residual volatiles evaporation were not studied in the present work.

## 2. Materials

**Figure 1:**
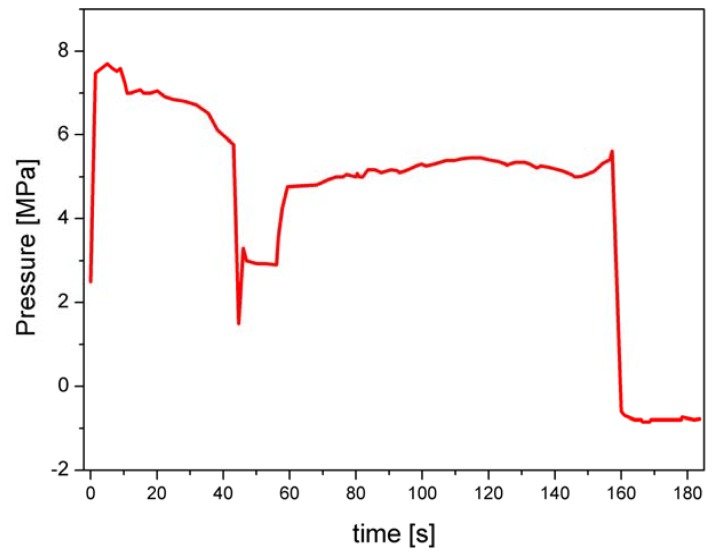
Pressure-time diagram of SMC moulding process (Formulation C) used for the calculation of optimal sampling conditions.

Samples of SMC were produced under defined temperature (148 °C), and different pressure (3.5 to 14 MPa) and moulding time (60 to 360 s) conditions. Plates having different glass fibres content (0%, 10%, 20% and 30%) were studied. SMC formulations are listed in Table 1. Figure 1 shows a pressure-time diagram for the formulation C, used to the calculation of the optimal conditions for the topographic characterisation.

**Table 1:** SMC Formulations used in the present study.

	wt.%			
	**A**	**B**	**C**	**D**
Unsaturated PES resin	16.6	15.0	13.3	11.7
Thermoplast	13.4	12.1	10.7	9.4
Thermoplastic solution in styrene	3.4	3.0	2.7	2.3
Initiator	0.4	0.3	0.3	0.3
Zinc stearate	1.0	0.9	0.8	0.7
Additives	0.8	0.7	0.6	0.5
Magnesium oxide	1.2	1.0	0.9	0.8
Calcium carbonate (filler)	63.2	56.9	50.6	44.3
Glass fibres, chopped	0.0	10.0	20.0	30.0

## 3. Experimental Section

### 3.1. Topography measurements 

A MicroGlider^®^ (FRT, Germany) imaging instrument was used for the optical analysis of the topography of SMC surfaces. Unlike conventional microscopy, which simultaneously images all the points in the field of view and captures a 2D image, the chromatic confocal microscope records only one object point per given unit of time. The field measured is reconstructed by *x-y* scanning. This novel optoelectronic setup, based on a quasi confocal, z-axis extended field, was developed for a high resolution non-contact 3D surface metrology, including roughness characterization and surface flaw detection.

**Figure 2:**
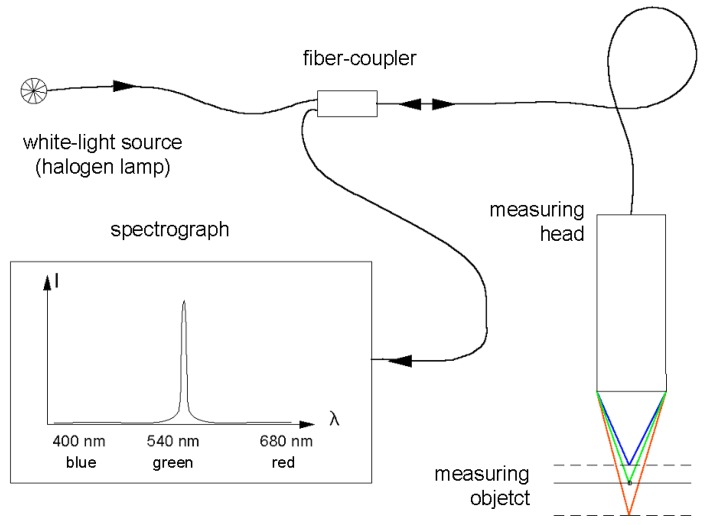
Schematic presentation of the measuring principle of chromatic confocal microscopy.

The instrument uses a chromatic white-light sensor (CWL), which is based on the principle of chromatic aberration of light [3]. As can be seen in Figure 2, white-light is focused on the surface by a measuring head with a strongly wavelength-dependent focal length (chromatic aberration). The spectrum of the light scattered on the surface generates a peak in the spectrometer. The wavelength of this peak along with a calibration table reveals the distance from sensor to sample. The sensor works on transparent, highly reflective or even matt black surfaces [4], it is extremely fast and has virtually no edge effects. 

The instrument allows a lateral measuring range and a vertical measuring range up to 100 mm and 380 µm, respectively, and a lateral resolution and vertical resolution up to 1 µm and 3 nm, respectively.

In [5,6], chromatic confocal microscopy was compared with high resolution scandisk confocal microscopy (SCDM). According to these studies, wider cut-off lengths and larger z-ranges make chromatic confocal imaging more appropriate than SCDM to measure topographical characteristics of composites. 

Depending on the SMC surface characteristics, other non-contact measuring methods (SEM, CLSM, CSOM, CSL, etc.) can be used. In order to obtain statistically representative topographical data a proper combination of cut-off length, z-range and resolution has to be used. It is important to note that a method with a very high resolution can be inadequate, if the available cut-off length or z-range is too small. On the other hand, the use of a very high resolution and larger cut-off lengths (scan areas) results in a high amount of data and extremely long calculation times which requires a special or not existent hardware and software.

### 3.2. Topographic characterisation parameters

**Figure 3:**
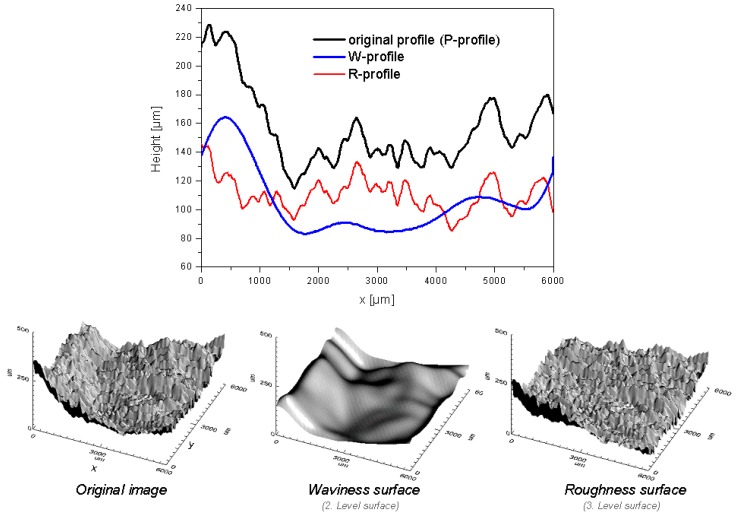
Above: W-profile and R-profile obtained applying FFT-filtering (original profile at y = 3,000 µm of the original image below). Below: waviness and roughness surfaces obtained applying FFT- filtering to the original topographic data.

To determine roughness and waviness of a 2D profile, Fast Fourier Transformation filtering is applied to separate the long-wavy and short-wavy profile components. By filtering a measured profile (primary data, P-profile), a roughness filtered profile (R-profile) and a waviness filtered profile (W-profile) are obtained (cf. Figure 3). The application of an equivalent mathematical procedure to 3D topographical data [9,10] results in the separation of waviness and roughness surfaces.

Wave height W_t_ is defined as the distance between the highest elevation and the deepest valley of the W-surface. Waviness W_z_ is obtained by the arithmetic average of 25 W_zi_ values of the W-surface, each one corresponding to the distance between the highest elevation and the deepest valley measured inside each one of 25 extracted sub-areas [9]. 

R_a_ is the arithmetic average of all distances between z-value and mean height plane inside a R-surface defined by M *x* N evaluated points:

(1) $$R_{a}=\frac{1}{MN}\sum_{j=1}^{M}\sum_{k=1}^{N}\;\left|z_{jk}-\bar{z}\right|$$

R_q_ is the square average of all distances between z-value and the mean height plane of the R-surface. The resulting value is quite bigger than *R_a_*:

(2) $$R_{q}=\sqrt{\begin{array}{cc} \frac{1}{MN} & \sum_{j=1}^{M}\sum_{k=1}^{N}{\left(z_{jk}-\bar{z}\right)}^{2} \end{array}}$$

R_z_ parameter commonly called ‘mean roughness’ is defined as the arithmetic average of twenty five R_zi_ values, each one corresponding to the distance between the highest elevation and the deepest valley measured inside each one of twenty five extracted sub-areas.

### 3.3. Calculation of optimal sampling conditions

Cut-off length (L_m_), defined as the length of one side of the square sampling area, and resolution (distance between measured points Δ_x_, assuming that Δ_x_ = Δ_y_) are the most important sampling parameters, which apart from particular instrumental dependent parameters, such as light intensity, measuring frequency, etc, have to be optimally defined before characterising any topography.

Tsukada and Sasajima [7] and Yim and Kim [8] have discussed the problem of an optimum sampling interval (L_m_) by checking the variance of the root mean square roughness (R_q_) for a surface under different sampling intervals. According to Stout *et al*. [9], a recommendation for the choice of sampling interval is doubtful because of the fact that optimum L_m_ seems to influence the amplitude parameters (wave height W_t_ and waviness W_z_). 

The use of tables that relate the mean rough height (R_z_), root mean square roughness (R_q_) and arithmetic mean roughness (R_a_) with L_m_ is frequently recommended to setting the optimal value of L_m_ for periodic as well as non-periodic surfaces.

As optimal sampling conditions are strongly dependent on the type of material to be characterized, researcher experience is usually required. For this reason, a systematic procedure to define optimal cut-off length and resolution values is proposed:

(1) Acquiring topographical data at the highest resolution available (minimal value of Δ_x_) using different L_m_ values. Here two different procedures are recommended:
  (a) only one measurement at the highest L_m_ and posterior zooming (sub-area extractions), or
  (b) independent measurements using the same zero point position.
(2) The use of statistical criteria in order to define an optimal value of L_m_ by analysing W_z_, R_z_ and R_a_ curves as functions of L_m_.
(3) Acquiring topographical data with the defined optimal L_m_ using different values of resolution.
(4) The use of statistical and topographical criteria to analyse R_z_ and R_a_ as functions of Δ_x_ , in order to define an optimal resolution.

**Figure 4:**
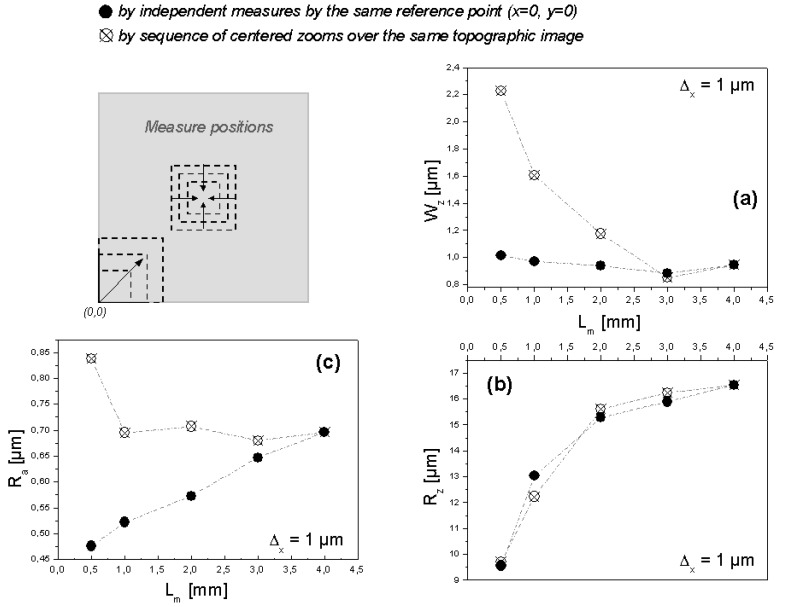
Topographic parameters of a SMC surface as a function of L_m_.

**Figure 5:**
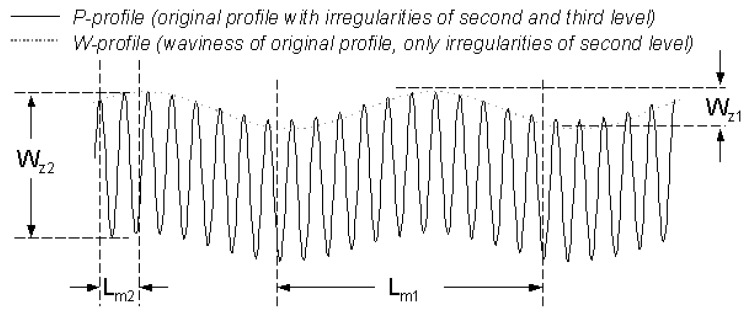
Dependency of wave height on cut-off length.

Applying the steps of the previous procedure to a SMC surface of formulation C (cf. Table 1), moulded under 5 MPa during 160 seconds, results first in the curves shown in Figure 4. Calculated waviness values of a SMC surface (Figure 4a) using a resolution of Δ_x_ = 1 µm, shows an important dependence on the cut-off lengths up to L_m_ = 3 mm. As a result of Fast Fourier Transformation (FFT) filtering operations [10] of the topographical data, the obtained waviness is always strongly dependent on measure scale. The calculation of the waviness of an idealized two dimensional P-profile (primary profile without mathematical corrections) showed in Figure 5, produces different wave height values depending on cut-off length. Using a longer cut-off length (L_m1_), the wave height (W_z1_) of the FFT-filtered W-profile quantifies the amplitude of second level (“waviness” according to DIN 4760) of the P-profile. But using a shorter cut-off length (L_m2_), FFT-filtering tends to quantify the amplitude of the irregularities of third level (“grooves” according to DIN 4760), which produces a higher value of wave height (W_z2_).

Depending on the type of surface and on the aim of the characterisation, it is important to consider the previous explanation in order to find and probe the optimal measure scale to describe the waviness. In the same way as W_z_, calculated values of R_z_ show an important dependency on L_m_ (Figure 4b) with a tendency to a constant value of 16.5 µm at about L_m_ = 3 mm. However, the velocity of this convergence is practically independent of measuring position, because the surface irregularities between z = 10 µm and z = 17 µm are regularly distributed over the surface. In other words, over L _m_ = 3 mm the measured value of R_z_ becomes statistically reliable.

Values of R_a_ are highly sensitive to measuring position (Figure 4c). By sub-area extractions, a clear tendency to a constant R_a_ value is observed if L_m_ > 1 mm because over the same region of the surface the irregularities of fourth level (DIN 4760), also called “scales” or “crusts” (z = 0.45 µm to 0.85 µm in this case), have almost the same height. To characterize this surface it is important to note that the mean height of “scales” and “crusts” depends on measuring region, probably due to two causes: the use of a not at all regular polished metallic mould during the production of the SMC samples and irregular glass fibres orientation.

Considering the behaviour of W_z_, and R_z_, the optimal L_m_ value for this type of surface is 3 mm, or longer if one wants to assure even more the statistical reliability of the measures. Results obtained by analyzing the dependence of R_a_ on measuring position (R_a_ is universally understood and provides a useful basis for benchmarking), show that the optimal characterization of this fourth level irregularities demands the study of the surface by different measuring positions and to consider the topography of the metallic mould, as well as glass fibre content and orientation. 

**Figure 6:**
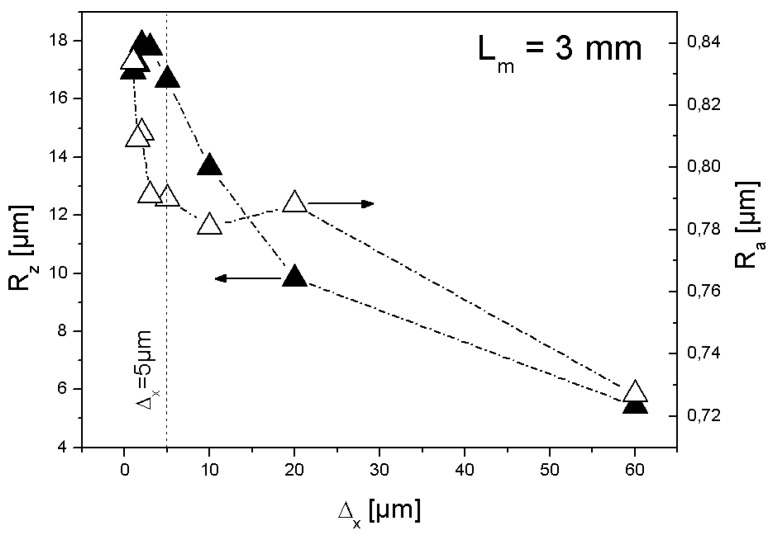
Roughness of the SMC topography as a function of resolution used.

Using the defined optimal cut-off length (L_m_ = 3 mm), new topographical data were obtained applying different values of resolution. Figure 6 shows the behaviour of calculated R_z_ and R_a_ as a function of resolution. The selection of the optimal resolution depends on two conditions: highest statistical reliability and shorter measurement time, which is specially important in the case of scanning microscopy such as chromatic confocal imaging. Refining resolution (use of smaller Δ_x_) means the measurement of more points in x- and y- direction and as a consequence longer measurement time (f*-*times more points means an increase of measurement time in a factor of at least f^2^).

**Figure 7:**
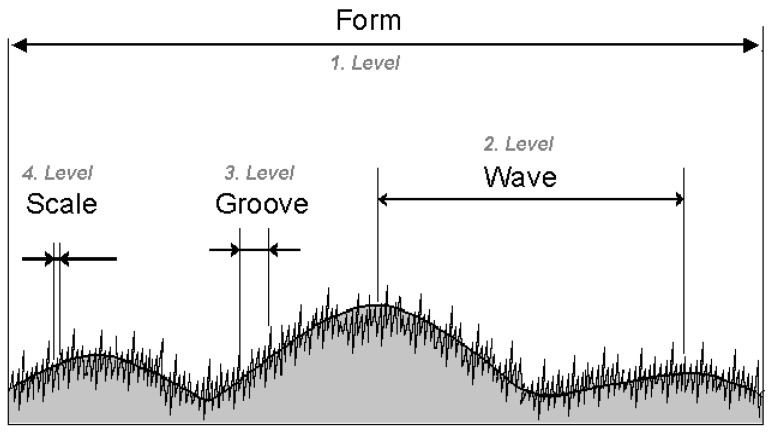
Graphic representation of the inhomogeneities of fine parameters up to four levels of a topographic profile according to DIN 4760.

From a statistical point of view, calculated R_z_ is reliable at resolutions finer than 5 µm. If the characterisation of the surface is oriented to the morphology of third level (“grooves” by DIN 4760) (cf. Figure 7), then Δ_x_ = 5 µm will be satisfactory. To characterise irregularities of fourth level the recommended lateral resolution is the finest available by the method used, in this case Δ_x_ = 1 µm. However, between resolutions of 5 µm and 1 µm the values of R_a_ show a difference of only 44 nanometers, about 5% of the measured arithmetic mean roughness at the finest resolution (R_a_ = 0.834 µm).

In order to probe the reliability of the optimized sampling conditions (L_m_ = 3 mm and Δ_x_ = 5 µm), seven independent topographic measurements were performed over different regions of the sample surface. Standard deviation of the resulting parameters (Table 2) show that R_a_ is statistically more reliable than W_z_ and R_z_. However, these results also show that the topographic parameters are dependent on the measurement position. The reasons will be analysed in the next paragraphs.

**Table 2:** Calculated parameters of seven independent measurements (different positions) on the same SMC plate (L_m_ = 3 mm, Δ_x_ = 5 µm).

Parameter	Mean value, µm	Standard deviation, µm
W_z_	0.95	0.242
R_z_	17.23	4.082
R_a_	0.76	0.066

### 3.4. Topographic characterization

**Figure 8:**
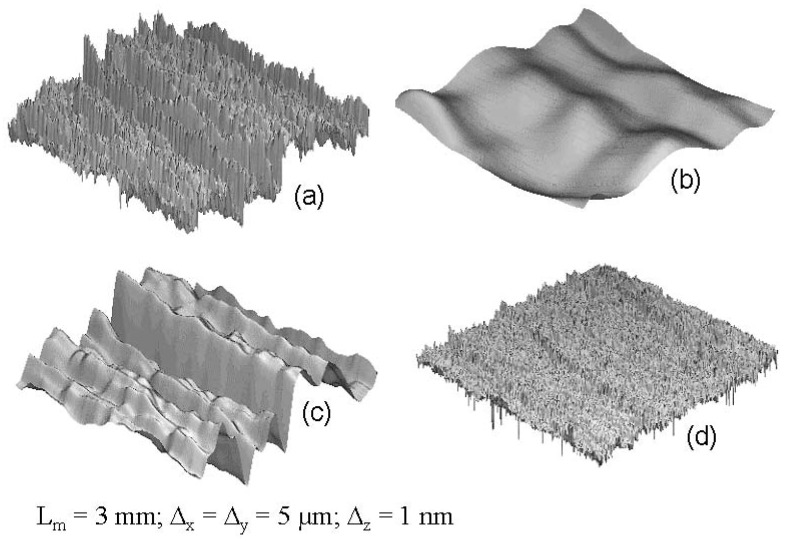
(a) Total SMC topography. (b) Isolated short waviness. (c) Isolated mean roughness. (d) Isolated arithmetic mean roughness.

Using the obtained optimal parameters, about 200 topographic measurements at the same and different coordinates of the SMC-plates of formulation C (cf. Table 1) were realized, corresponding to identified positions of the metallic mould. The resulting surface data were processed by FRT-Mark III Software (FRT, Germany) using mathematical filtering by Fast Fourier Transformation Method, whose graphical 3D representation can be seen in Figure 8. Three meso-topographic parameters were calculated: short-waviness (W_z_), mean-roughness (R_z_) and number of long waves on L_m_ (N_z_). Three micro-topographic parameters were obtained: arithmetic mean roughness (R_a_), number of short waves on L_m_ (N_a_), and porosity (V_o_), defined as the filling quantity or void volume under the mean height of the surface per area unit. Additionally, long-waviness (L-W_z_) using L_m_ = 100 mm and Δ_x_ = 100 µm was measured in order to study the macro-morphology of the surface.

## 4. Results and Discussion 

### 4.1. Moulding conditions and topographical transfer from metallic mould to SMC surface

Schubel [11] used microscopic and stylus profiling methods in order to correlate R_a_ values to study roughness effects on SMC surface quality. To our present work, three different SMC plates that correpond to defined moulding conditions (pressure and moulding time) were selected. On each one of them, the topography of sixty defined positions were characterised by chromatic confocal imaging using L_m_ = 3 mm and Δ_x_ = 5 µm. 

**Figure 9:**
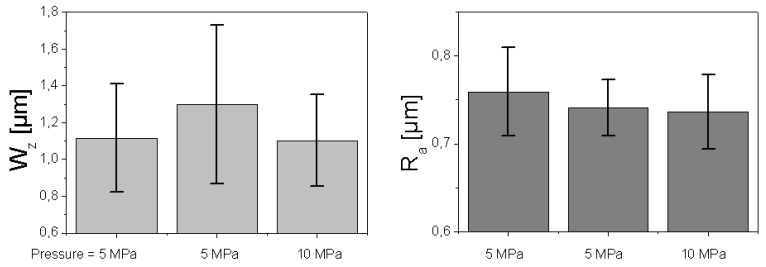

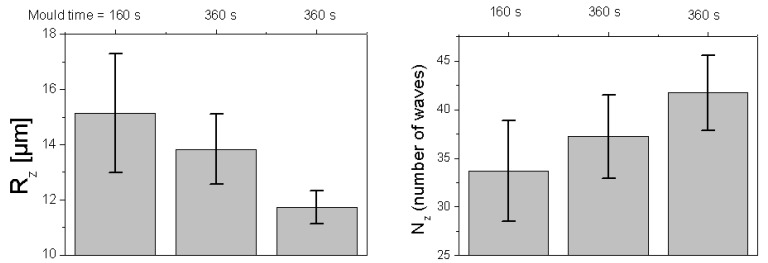
Influence of moulding conditions (pressure, moulding time) on meso (W_z_, R_z_, N_z_) and micro topography (R_a_).

Figure 9 shows that, from a statistical point of view, mean values of W_z_, R_z_ and N_z_ could characterise the topography as a function of moulding conditions. According to our results, moulding conditions control the meso-topography of the SMC surfaces studied and obviously not the micro-topography characterised by R_a_ values.

**Figure 10:**
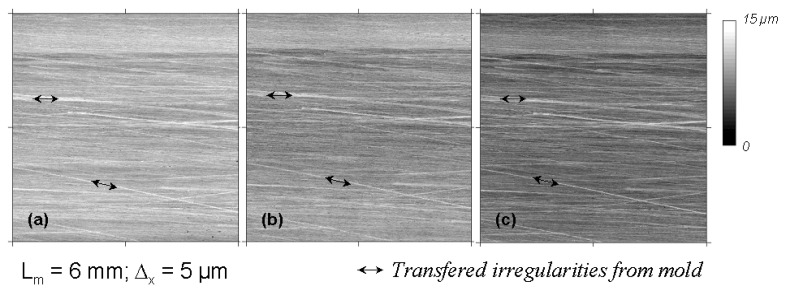
Transference of mould grooves defines the meso-topography of third level (DIN 4760) of SMC surfaces. Moulding conditions: (a) 5 MPa and 160s). (b) 5 MPa and 360s. **(c)** 10 MPa and 360s.

By comparing the measured topographic parameters between plates at the same position (Figure 10), it is clear that the topography of the metallic mould controls the resultant meso-topography of the SMC surface in a more specific way than moulding conditions, by transference of its surface irregularities or third level (grooves). 

**Figure 11:**
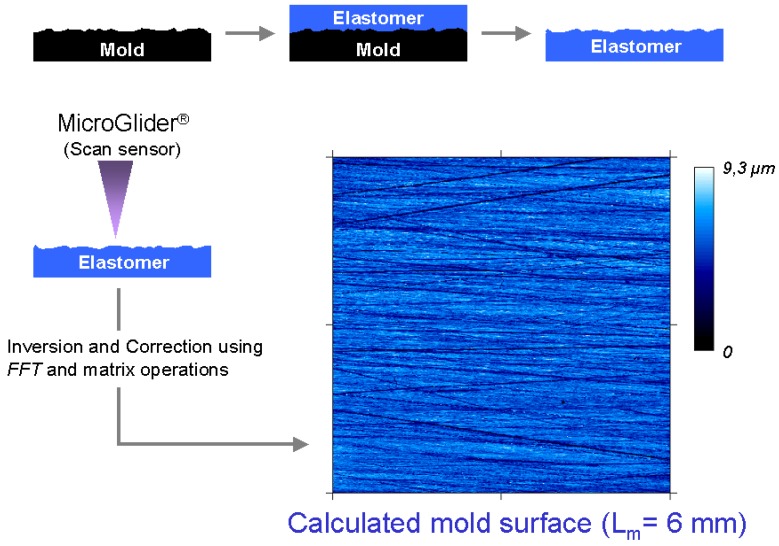
Indirect calculation of mould topography by transfer in silicon elastomer.

As a first important conclusion, by the SMC surfaces studied, any topographic characterisation of surface modification processes (cleaning by powerwash for example) must be realized by comparing topographical changes of identified mould-plate positions. For a systematic study of mould influence on resultant SMC surfaces, it is necessary to previously measure the topography of the metallic mould. However, a direct measure of the mould topography was not possible because the size and weight of the metal piece. For this reason, a print of the mould surface was obtained using soft moulding silicon elastomer (Figure 11), which is proved to reproduce nanoscale structures with great fidelity [12]. Before applying this process to the metallic mould, an experimental probe and calibration of this procedure were realized by measuring the topography of a small metallic piece of the same material of the mould and comparing its topography with the topography of the obtained elastomer surface.

**Figure 12:**
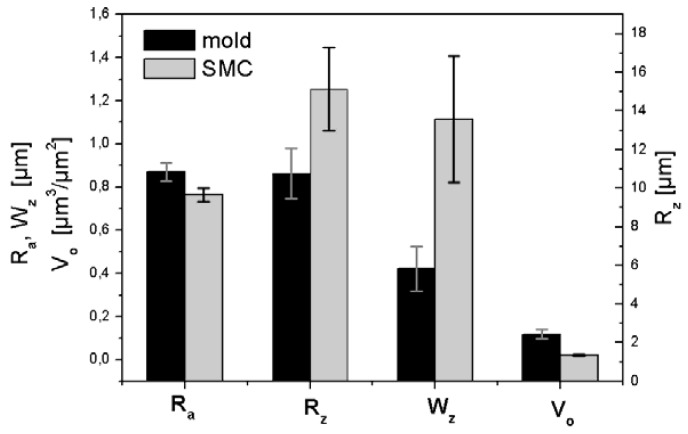
Topographic transfer from metallic mold to SMC surface.

According to our results, the transference of micro-topographic irregularities (R_a_) from mould to SMC surface is relatively low (Figure 12). The calculation of Wenzel roughness factor [13] (the ratio between the real surface area and the geometric projected area) shows that the mould effective area is about only 1% bigger than the effective area of SMC surface by using the optimal resolution of 5 µm. 

**Figure 13:**
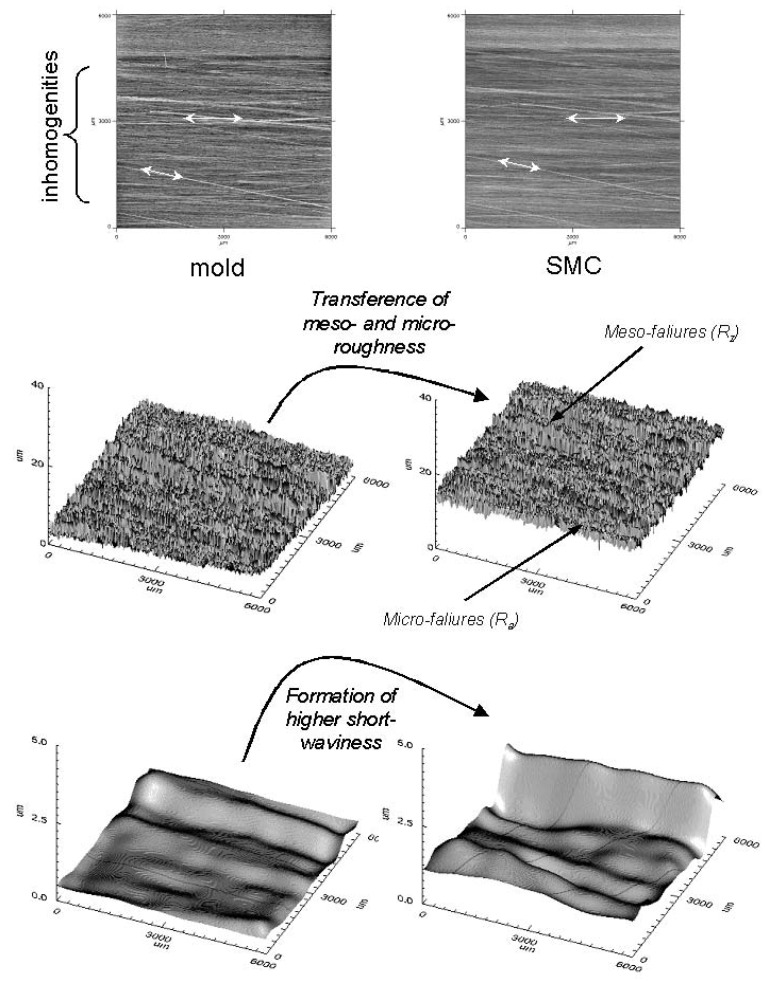
Transfer of meso- and micro morphologies from mould to SMC surface.

However, the transference of meso-topography, characterised as R_z_ is strong, principally because a notable increase of the short waviness from mould to SMC surface, presumably due to adhesion during separation (Figure 13), that could be responsible for a “shrinkage effect” of 2D-profiles observed by Schubel *et al*. [11] and attributed to mould surface and to a volumetric resin shrinkage. 

**Figure 14:**
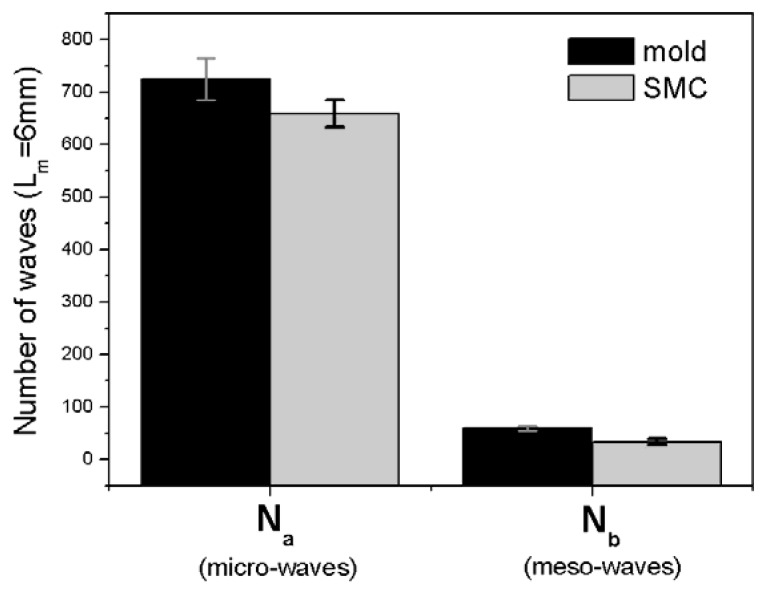
Meso- and micro-waves transference from mould to SMC surface.

Transfer of meso-waves (N_z_) is only about 57% (Figure 14), but the transference of their height (characterized as R_z_) is about is about 141% due to the notable increase of short waviness after moulding (cf. Figure 12).

**Figure 15:**
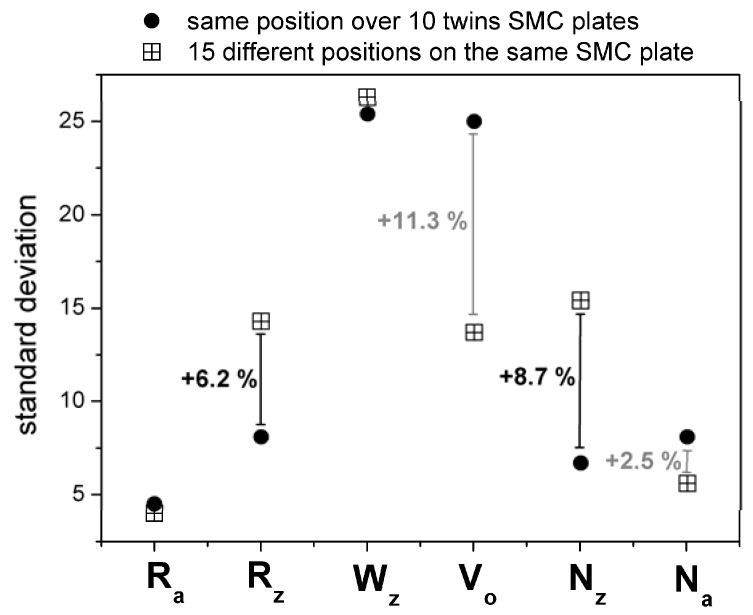
Impact of sampling (positioning) criteria on statistical reliability.

After measuring 15 identified positions over 10 different twin SMC plates (produced with the same moulding conditions: 5 MPa, 160s), it was possible to compare the influence of mould topography on the statistical reliability of the topographic parameters. Figure 15 shows that mould topography influences strongly the SMC meso-topography (R_z_ and N_z_ varies notably from position to position). By fixing a position over 10 twin plates, R_z_ and N_z_ are statistically more reliable. Only the effect of glass fibres orientation (prepreg orientation) reflected in porosity (V_o_) seems to control the meso and probably micro-topography of each measured area. 

### 4.2. Effect of glass fiber content on microtopography

Le [14] studied the internal microstructure and porosity of SMC. As we have seen in the last section, orientation and content of glass fibres influence not only the internal structure, but also the micro-topography of the SMC surface. In order to study the effect of glass fibres, the topography of the four different recipes of SMC plates containing 0, 10%, 20% and 30% of glass fibres (cf. Table 1) were analyzed.

**Figure 16:**
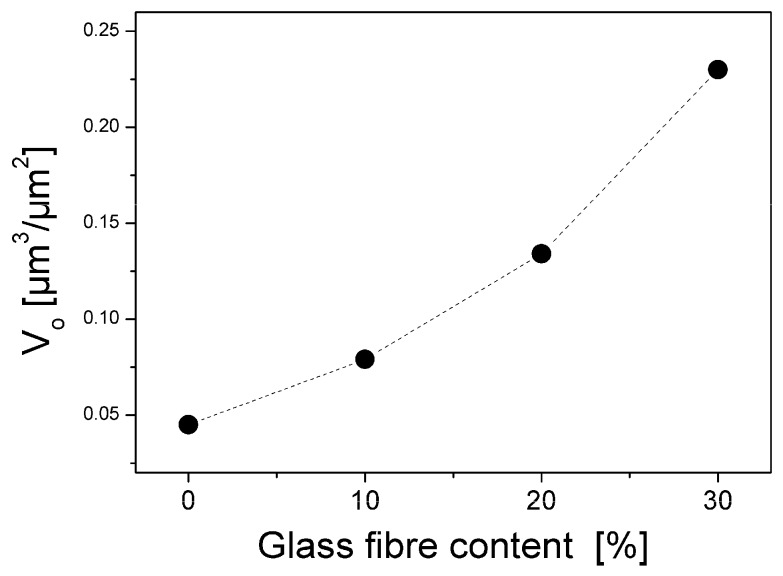
Effect of glass fibre content on porosity. Before calculation of Porosity (V_o_), short waviness was filtrated (removed). The values of Porosity in this graphic are not influenced by short waviness.

**Figure 17:**
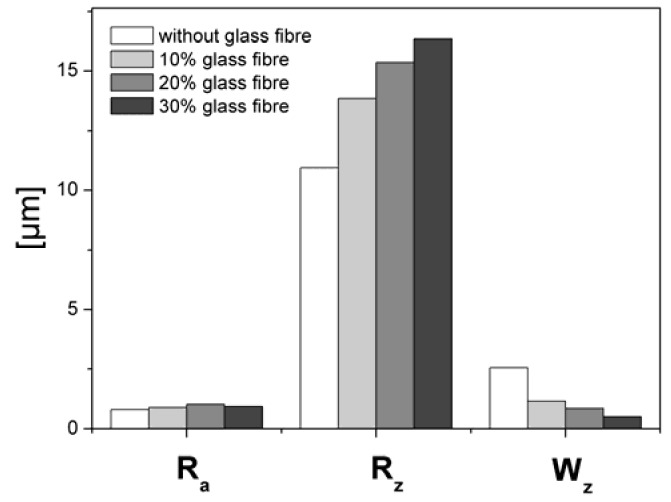
Effect of glass fibre content on topographic parameters.

According to our results, the glass fibres increase the porosity of the studied SMC surfaces (Figure 16). A detailed study shows (Figure 17) that the micro-topography (characterized as R_a_) is practically not influenced by the glass fibre content. In this case, the meso-topography was modified by increasing of R_z _ and as a consequence V_o_ (cf. Figure 16). Decrease of short waviness is a result of an increase of viscosity of prepreg during moulding, cooling, drying and shrinkage, and especially of a decrease of adhesion between mould and SMC surface during separation, presumably due to the presence of glass fibres.

### 4.3. Influence of moulding conditions on the long and short waviness

**Figure 18:**
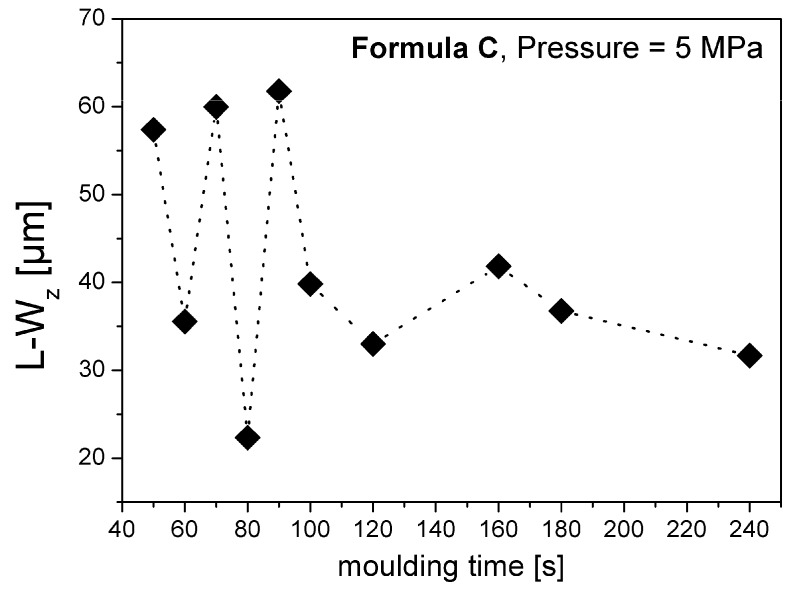
Effect of moulding time on long waviness of recipe C.

Surface long waviness (L-W_z_) values of the four different formulations of SMC materials produced by different conditions (pressure, moulding time) were measured using L_m_ = 100 mm and Δ _x_= 100 µm. In general, a stabilization of long waviness with the increase of moulding time was observed. Figure 18 shows the special case of formulation C. 

**Figure 19:**
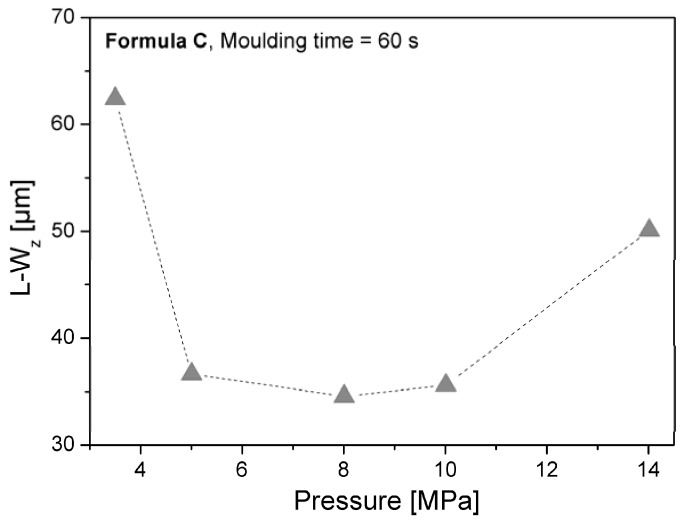
Effect of moulding pressure on long waviness at L_m_ = 100 mm and Δ_x_ = 100 µm.

**Figure 20:**
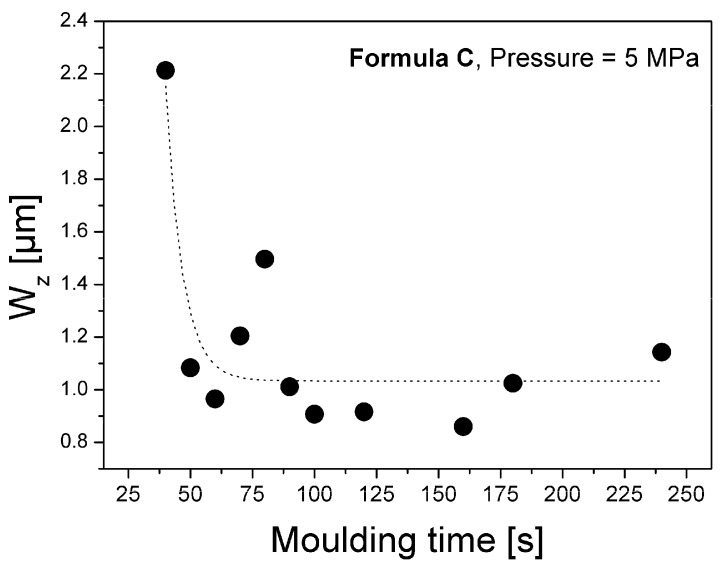
Decrease of short waviness by moulding time.

According to our results, pressure applied during moulding controls the resulting long waviness of the SMC recipes. Figure 19 shows the behaviour of long waviness as a function of pressure applied after 60 seconds moulding of formulation C. Pressure of 8 MPa leads to a minimum of long waviness. Short waviness W_z_, decreases with longer moulding time, as shown in Figure 20.

### 4.4. Effect of prepregs placement on macro- and meso-morphology

**Figure 21:**
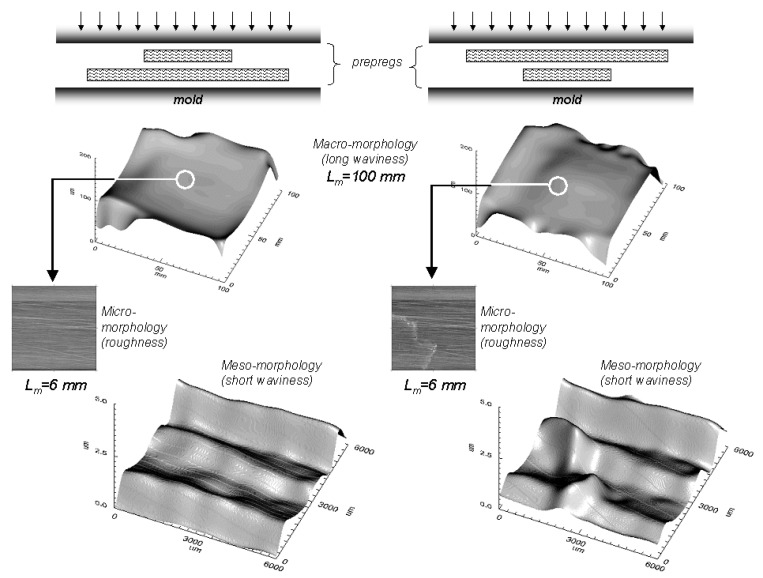
Effect of prepregs placement procedure on macro-, meso- and micro-morphologies.

It was found that the placement procedure of prepregs influences not only the long waviness (macro-morphology) of the resulting SMC surface, but also the short waviness (meso-morphology). Figure 21 schematizes two different placement procedures of prepregs before moulding and their effect on the resulting macro-morphology (L_m_ = 100 mm) of the SMC surfaces. In the case of the second placement procedure, a meso-morphologic failure is produced on the resulting surface, which leads to an important effect on measured short waviness.

## 4. Conclusions 

**Figure 22:**
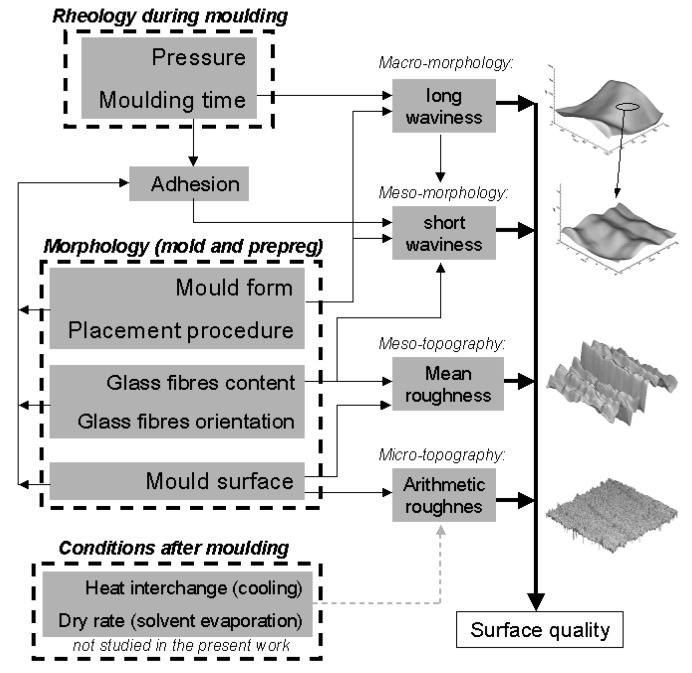
Schematization of the influence of production variables on the resulting topography of SMC surfaces (temperature and chemical composition are considered to be constant) for the SMC formulations studied.

The topographical study of SMC materials using a length scale concept allows to characterise surfaces separately by considering and analysing their specific morphologies caused by moulding conditions, metallic mould topography, glass fibre content and glass fibre orientation. With the resulting information, the correlation of topography and processing conditions is more specific and explanatory. As an example of application, the proposed topographic characterisation at different length scales for the studied SMC formulations and production conditions, leads to following conclusions (see Figure 22):

- At a constant temperature, rheology during moulding, controlled by pressure and moulding time, defines the resulting long waviness (macro-morphology studied by L_m_ = 100 mm), which is simultaneously controlled by the mould form and prepregs placement procedure.
- Mould morphology and prepregs placement also control short waviness (meso-morphology studied by L_m_ = 3 mm) directly and due to adhesion between mould and SMC surface during detaching.
- Orientation and size of glass fibres can affect the short waviness through adhesion during detaching, but in general control the meso topography (characterized as R_z_).
- Mould surface defines always the meso topography (R_z_) but, depending on mould inhomogeneities can also influence on the micro topography (characterized as R_a_).
